# Immunogenicity after vaccination of COVID-19 vaccines in patients with cancer: a prospective, single center, observational study

**DOI:** 10.1007/s10147-024-02470-x

**Published:** 2024-02-21

**Authors:** Yuki Katsuya, Tatsuya Yoshida, Atsuo Takashima, Kan Yonemori, Akihiro Ohba, Shu Yazaki, Shigehiro Yagishita, Hiroko Nakahama, Osamu Kobayashi, Masatoshi Yanagida, Yasuhiro Irino, Akinobu Hamada, Noboru Yamamoto

**Affiliations:** 1 Department of Experimental Therapeutics, National Cancer Center Hospital, 5-1-1 Tsukiji, Chuo-ku, Tokyo, 1050045 Japan; 2 Department of Thoracic Oncology, National Cancer Center Hospital, 5-1-1 Tsukiji, Chuo-ku, Tokyo, 1050045 Japan; 3 Department of Gastrointestinal Medical Oncology, National Cancer Center Hospital, 5-1-1 Tsukiji, Chuo-ku, Tokyo, 1050045 Japan; 4 Department of Medical Oncology, National Cancer Center Hospital, 5-1-1 Tsukiji, Chuo-ku, Tokyo, 1050045 Japan; 5 Department of Hepatobiliary and Pancreatic Oncology, National Cancer Center Hospital, 5-1-1 Tsukiji, Chuo-ku, Tokyo, 1050045 Japan; 6 Division of Molecular Pharmacology, National Cancer Center Hospital, 5-1-1 Tsukiji, Chuo-ku, Tokyo, 1050045 Japan; 7 Department of Nursing, National Cancer Center Hospital, 5-1-1 Tsukiji, Chuo-ku, Tokyo, 1050045 Japan; 8 Department of Infectious Diseases, National Cancer Center Hospital, 5-1-1 Tsukiji, Chuo-ku, Tokyo, 1050045 Japan; 9 Applied Diagnostic Research Group, Central Research Laboratories, Sysmex Corporation, 4-4-4 Takatsukadai, Nishi-ku, Kobe, 651-2271 Japan

**Keywords:** SARS-CoV-2, Cancer patients, Cancer treatments, Immunogenicity, COVID-19 vaccine

## Abstract

**Background:**

Patients with cancer, particularly those undergoing chemotherapy, are at risk from the low immunogenicity of Coronavirus Disease 19 (COVID-19) vaccines.

**Methods:**

This prospective study assessed the seroconversion rate of COVID-19 vaccines among patients with cancer and hospital staff. Severe acute respiratory syndrome coronavirus 2 (SARS-CoV-2) spike protein-specific IgG (S-IgG) concentrations were evaluated before the first vaccination, and 1–3 and 4–6 months after the second vaccination. The primary endpoint was the seroconversion rate measured 1–3 months after the second vaccine.

**Results:**

In total, 590 patients and 183 healthy hospital staff were analyzed. At 1–3 months after the second vaccination, the S-IgG antibody concentration exceeded the cut-off value (20 BAU/mL) in 96.1% (567/590) of the patients with cancer and 100% (183/183) of the healthy controls (*p* = 0.0024). At 4–6 months after the second vaccination, the S-IgG antibody concentration exceeded the cut-off value (20 BAU/ml for S-IgG) in 93.1% (461/495) of the patients with cancer and 100% (170/170) of the healthy controls (*p* < 0.0001). Old age, being male, and low lymphocyte count were related to low SARS-CoV-2 S-IgG levels 1–3 months after the second vaccination among patients, while body mass index, smoking history, and serum albumin level were not. Patients undergoing platinum combination therapy and alkylating agent among cytotoxic drugs, and PARP inhibitor, mTOR inhibitor, and BCR-ABL inhibitor exhibited a low S-IgG antibody concentration compared to the no treatment group.

**Conclusions:**

COVID-19 vaccine immunogenicity was reduced among patients with cancer, especially under several treatment regimens.

## Introduction

Patients with cancer report high morbidity and mortality rates associated with Coronavirus Disease 19 (COVID-19), which is caused by severe acute respiratory syndrome coronavirus 2 (SARS-CoV-2) [1, 2]. Before vaccination, the difference between the SARS-CoV-2 antibody status of patients with cancer and health care workers in Japan was reported. Although the seroprevalence was approximately 1% among both patients and health care workers, the nucleocapsid protein-specific IgG (N-IgG) and spike protein-specific IgG (S-IgG) serum antibody concentrations were significantly lower in patients with cancer than in health care workers [3]. In contrast, another study based on SARS-CoV-2 serological screening suggested limitation of serological tests to detect SARS-CoV-2 infection compared to the RT-PCR method [4].

As of April 5, 2021, there were more than 135 million confirmed COVID-19 cases with 3 million deaths worldwide, and 506,284 confirmed COVID-19 cases with 9382 deaths in Japan [5]. Novel COVID-19 vaccines such as BNT162b2 (Pfizer-BioNTech) and mRNA-1273 (Moderna) were quickly developed and recommended for patients with cancer to prevent severe illness, hospitalization, or death. In Japan, the vaccination of healthcare workers nationwide was commenced on February 2021 and that of older adults and patients with comorbidities on April 2021.

Vaccination strategy is quite important in both health care workers and patients with tumors. Facing unprecedented crises, numerous communities had also set up registries and observational studies to collect data on COVID-19 among patients with cancer. Previous studies showed that even patients with solid tumors under treatment develop a satisfactory immune response to COVID-19 vaccines [6]; however, their seroconversion rate and IgG titers were lower than those of the general population [3, 4]. Patients with solid tumors receiving cytotoxic chemotherapy are at risk from the low immunogenicity of COVID-19 vaccines but are not at significant risk from immune checkpoint inhibitors [8]. However, the vaccine-induced immune response in targeted therapy has not been clarified. In addition, data from very few Asian patients with cancer are available [9], and data on the change of antibody titers in the long term has been insufficient.

We conducted a prospective study to assess the immunogenicity of original COVID-19 vaccines over a long period in patients with cancer in Japan. We also assessed whether treatment for cancer, including chemotherapy and immune checkpoint inhibitors, influences the immune response to COVID-19 vaccines.

## Methods

### Study design and participants

This is a prospective, single-center, observational study conducted at the National Cancer Center Hospital in Japan. We enrolled participants from two groups: patients and hospital staff. Patients aged ≥ 16 years and with cancer (mainly solid tumors but those with hematological malignancy were also enrolled) regardless of stage, histology, and treatment, that were taking blood tests at least every 3 months were eligible. Individuals working at National Cancer Center Hospital taking or planning to take COVID-19 vaccines were eligible, regardless of complications. Considering the effect of complications such as hypertension, diabetes, cancer, and autoimmune diseases on immunogenicity, hospital staff without complications were referred to as “healthy controls”.

All participants provided written informed consent. The study was conducted in accordance with the principles of the Declaration of Helsinki, Good Clinical Practice guidelines, and applicable government regulations. The study protocol was approved by the ethics committee of the National Cancer Center Hospital (UMIN000049403, UMIN000049430).

### Procedures

Participants received two doses of the COVID-19 vaccine (BNT162b2 of Pfizer-BioNTech, mRNA-1273 of Moderna, or others, including AZD1222 of AstraZeneca) as locally prescribed. The vaccination schedule in Japan was the same as the one in the US and European countries. During the study period, mRNA vaccines, such as BNT162b2 and mRNA-1273, accounted for 99.99% of the market in Japan.

Qualified healthcare workers drew blood samples by venipuncture at the National Cancer Center Hospital. Blood samples for the measurement of SARS-CoV-2 S-IgG, spike protein-specific IgM (S-IgM), and N-IgG serum antibody concentrations were collected immediately before the first dose of the vaccine was administered, and then 1–3 and 4–6 months after the second dose vaccination. Properly stored frozen serum was used for retrospective measurement and to replace blood drawing to reduce the strain on patients. Secondary use of measurement data from a previous study [10] was allowed according to the protocol because vaccination for hospital staff had started before this study.

S-IgG, S-IgM, and N-IgG concentrations were measured (Sysmex, Japan) as described in a previous study [11]. Cut-off values were 20 BAU/ml for S-IgG, 20 SU/ml for S-IgM, and 10 SU/ml for N-IgG; antibody concentrations exceeding the cut-off value were regarded as “positive”. According to the study, cutoff values were established as antibody concentrations that define acquired immunity, as assessed using serum samples from SARS-CoV-2 infected and uninfected patients. N-IgG was measured considering clinical and subclinical infection rate. S-IgM was measured to determine whether S-IgG levels were not sufficiently elevated, because there was a delay in seroconversion, or because immunity was not initially acquired.

A questionnaire administered to patients 1–3 months after the second vaccination was used to collect information regarding adverse events and COVID-19 infection. Laboratory data before vaccination, and follow-up data were collected via a chart review. For hospital staff, a questionnaire administered 1–3 months after the second vaccination was used to collect information regarding adverse events, and a questionnaire administered 12 months after the second vaccination was used to collect information about the incidence of COVID-19.

### Outcomes

The primary endpoint was the SARS-CoV-2 seroconversion rate (S-IgG positivity) measured 1–3 months after the second vaccination among patients with cancer and the healthy control of the hospital staff.

Secondary endpoints were the SARS-CoV-2 seroconversion rates measured at timepoints other than the primary endpoint. We also evaluated the impact of patient characteristics, laboratory data, types of vaccines, comorbid cancer, different cancer types, and treatment types on immunogenicity for SARS-CoV-2 vaccines.

### Statistical analysis

All participants with data available for 1–3 months after the second vaccination were included in immunogenicity analyses. Participants were categorized as patients with cancer and healthy controls, and data were analyzed at certain timepoints: pre-vaccination, 1–3 and 4–6 months after the second vaccination. Continuous variables are reported as medians (with 95% confidence intervals [CI]) and compared using the Mann–Whitney U test. Categorical variables are reported as numbers and percentages and compared using Fisher’s test. The significance threshold for *p*-values was less than 0.05 after correction for multiple comparisons by the Bonferroni method. Statistical analysis was performed using GraphPad Prism version 9.3 (GraphPad Software, San Diego, CA).

## Results

### Participant characteristics

**Fig. 1:**
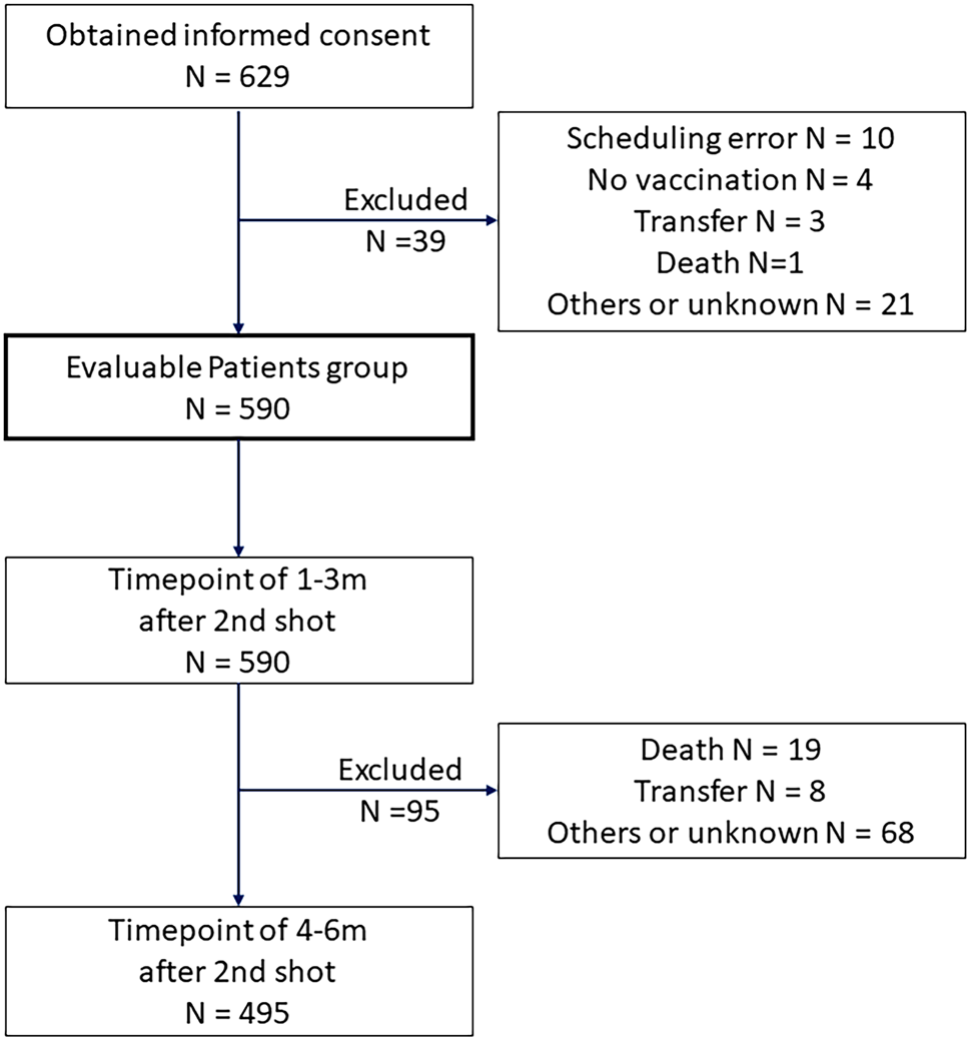
STROBE flow chart of the patients with cancer participants

**Fig. 2:**
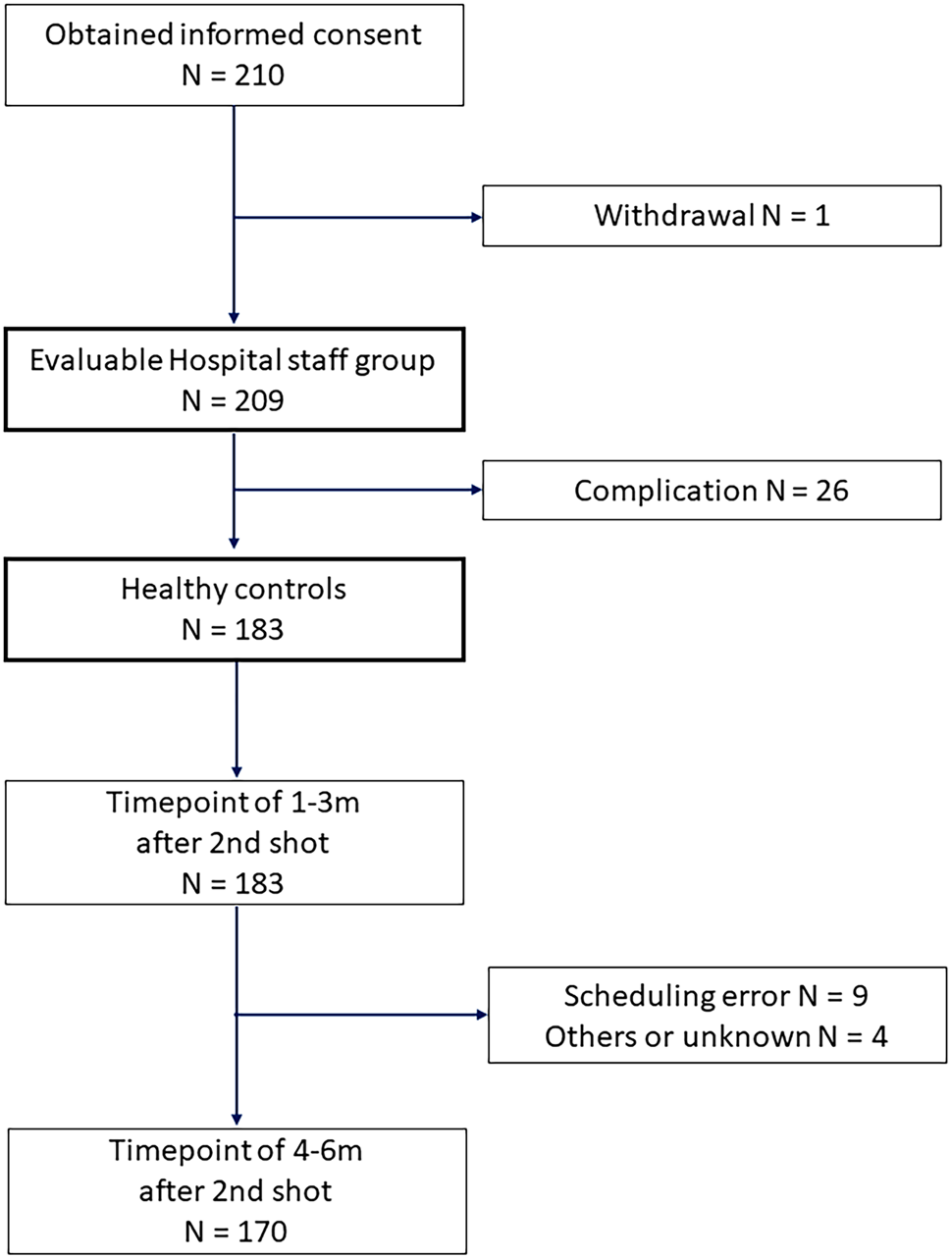
STROBE flow chart of the hospital staff participants

During April 5, 2021 to November 30, 2021, 629 patients with cancer (625 with solid tumors and 4 hematological tumors) and 210 hospital staff consented to participate in the study (Figs. 1, 2). Participants without blood test results 1–3 months after the second vaccination (39 patients including 10 affected by scheduling errors, 4 not-vaccinated-yet, 4 dead or transferred, and 21 for unknown reasons; and 1 hospital staff withdrawal) were excluded, 590 patients with cancer and 209 hospital staff were evaluable. In total, 183 hospital staff were regarded as healthy controls because 26 participants among the hospital staff had complications such as hypertension, diabetes, cancer, and autoimmune disease. Blood test results were available for 537 patients with cancer and 90 healthy controls before the first vaccination, and for 495 patients and 170 healthy controls 4–6 months after the second vaccination. The characteristics of the participants are summarized in Table 1. The median age was 66 and 45 among patients and hospital staff, respectively. The ratios of female participants were 81.8% and 54.6% among hospital staff and patients, respectively. At least 86.1% of patients and all hospital staff took mRNA vaccines (BNT162b2 or mRNA-1273). Eighteen patients underwent an operation, 3 underwent radiotherapy, 485 were administered drug treatment, and 85 did not undergo any treatment for cancer within the month preceding the first vaccination. The median time from the second vaccination to the blood test for the primary endpoint (1–3 months after second vaccination) was 41 days (min 16–max 109) for the patients and 61 days (min 31–max 107) for the healthy control group.

**Table 1:** Patient characteristic

	Patient	Hospital staff
**Total**	590	209
Age(median)	19–87 (66)	21–64 (45)
Sex		
M	268	38
F	322	172
BMI (median)	14.5–40.2 (23.0)	15.2–36.7 (21.5)
BMI over 30	19	10
BMI less than 18.5	64	23
Smoking history		
Never	255	165
Ex	233	39
Current	16	5
NA	85	0
ECOG PS		
0	357	
1	219	
2	5	
NA	8	
Vaccination		
BNT162b2	458	208
mRNA-1273	49	1
unknown	82	0
Complication		
Total	26	
Hypertension	16	
Diabetes	2	
Cancer	6	
Autoimmune disease	3	
Cancer type		
Non-small cell carcinoma	168	
Breast cancer	92	
Colorectal cancer	74	
Pancreas cancer	52	
Ovarian cancer	31	
Gastric cancer	20	
CNS tumor	17	
Bile duct tumor	15	
Melanoma	12	
Carcinoma of unknown primary	12	
Small cell carcinoma	10	
Thymic carcinoma	7	
Endometrial cancer	6	
Cervical cancer	6	
Hematologic tumor	4	
Others	64^a^	
Treatment within one months before 1st vaccination		
Operation	18	
Radiotherapy	3	
Drug treatment	485	
Cytotoxic drug, and antibody drug conjugate	231	
Small molecule targeted drug	103	
Immune checkpoint inhibitor	52	
Endocrine therapy with or without small molecule targeted drug	47	
Immune checkpoint inhibitor + cytotoxic drug	20	
Antibody	18	
Other	13	
No treatment	85	

^a^64 other tumors were as follows: 5 of Gastrointestinal Stromal Tumor (3 from small intestine), 4 of Neuroendocrine tumor, 3 of adenoid cystic carcinoma, 3 of duodenal cancer, 3 of carcinoid, 3 of angiosarcoma, 3 of leiomyosarcoma, 3 of liposarcoma, 2 of small intestine cancer, 2 of esophageal cancer, 2 of malignant pleural mesothelioma, 2 of appendix cancer, 2 of thyroid cancer, 2 of fallopian tube cancer, 2 of rhabdomyosarcoma, 2 of desmoid tumor, 1 of hepatocellular carcinoma, 1 of basal cell carcinoma, 1 of thymoma, 1 of gastroesophageal junction cancer, 1 of parathyroid cancer, 1 of enamel epithelioma, 1 of parotid cancer, 1 of duodenal papilla cancer, 1 of urothelial cancer, 1 of neuroendocrine carcinoma, 1 of extramammary Paget, 1 of buccal mucosa cancer, 1 of anal cancer, 1 of germ cell tumor, 1 of yolk sac tumor, 1 of spindle cell carcinoma, 1 of CIC-rearrangement sarcoma, 1 of synovial sarcoma, 1 of uterine carcinosarcoma, 1 of uterine sarcoma, 1 of fibromyxosarcoma

### SARS-CoV-2 seroconversion rate (S-IgG positivity) at each timepoint

**Fig. 3:**
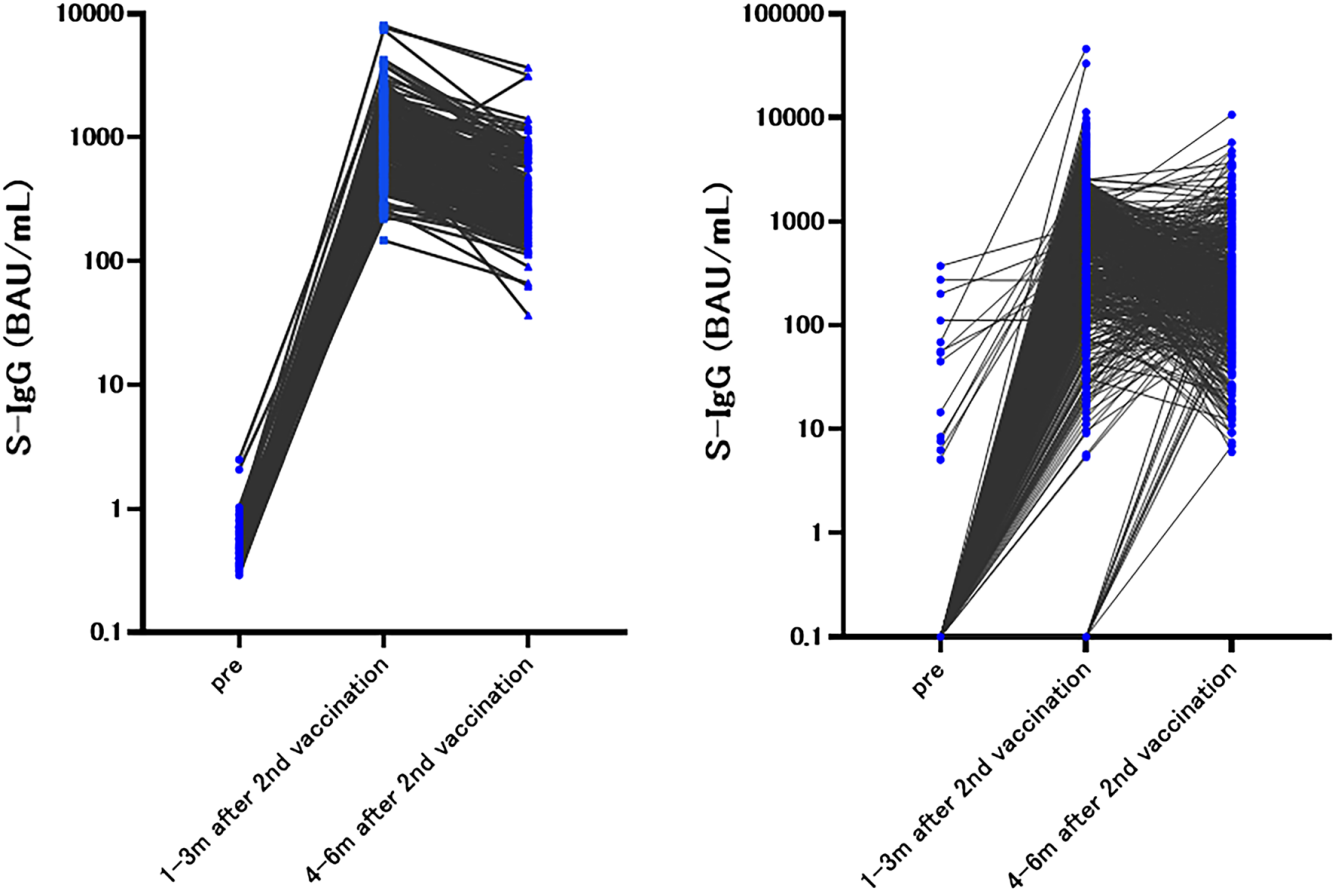
S-IgG concentration trend of pre-vaccination, 1–3 and 4–6 months after the second vaccination among the healthy controls (left)  and the patients (right)

At baseline, 1.4% (8/537) of patients had N-IgG antibody concentrations that exceeded the cut-off value (10 SU/mL), indicating past infection history of COVID-19. Five out of the seven patients declared a history of COVID-19 on a questionnaire, but two patients were not aware of the infection. None (0/90) of the healthy controls showed higher levels of N-IgG than the cut-off value (Table 2, Fig. 3).

**Table 2:** S-IgG concentration and positive rate of pre-vaccination, 1–3 and 4–6 months after the second vaccination among the patients and healthy controls

Patient	Pre	1–3 m after 2nd vaccination	4–6 m after 2nd vaccination
S-IgG*	8/537	567/590	461/495
Positive rate	1.4%	96.1%	93.1%
Median (95% CI)	0 (0.0–0.0)	761.8 (681.2–864.3)	198.3 (176.6–226.9)
N-IgG	7/537	6/590	4/495
Positive rate	1.3%	1.0%	0.8%
Median (95% CI)	0 (0.0–0.0)	0 (0.0–0.0)	0 (0.0–0.0)
S-IgM	2/537	191/590	12/495
Positive rate	0.0%	32.3%	2.4%
Median (95% CI)	0.6 (0.5–0.6)	9.8 (8.6–12.0)	1.4 (1.2–1.6)
Healthy control	pre	1-3 m after 2nd vaccination	4-6 m after 2nd vaccination
S-IgG*	0/90	183/183	170/170
Positive rate	0.0%	100%	100%
Median (95% CI)	0.49 (0.45–0.43)	920.8 (834.5–1107)	288.1 (257.5–319.7)
N-IgG	0/90	1/183	2/170
Positive rate	0.0%	0.5%	1.1%
Median (95% CI)	0.024 (0.02–0.029)	0.012 (0.0–0.018)	0 (0.0–0.0)
S-IgM	1/90	46/183	7/170
Positive rate	1.0%	25%	4.1%
Median (95% CI)	0.4 (0.32–0.52)	8.5 (6.84–10.8)	2.3 (2.0–2.7)
Fisher’s test of S-IgG*		0.0024	< 0.0001

At 1–3 months after the second vaccination, S-IgG antibody concentration exceeding the cut-off value (20 BAU/mL) was identified in 96.1% (567/590) of the patients and 100% (183/183) of the healthy controls (*p* = 0.0024). The S-IgG concentration among patients (median 761.8 BAU/mL, 95% CI of 681.2 to 864.3) was lower than that among healthy controls (median 920.8 BAU/mL, 95% CI of 834.5 to 1107). All 23 seronegative patients received drug therapies; 15 cytotoxic chemotherapy, 5 small molecule targeted drugs (including one combining endocrine therapy), 2 immune checkpoint inhibitor, and 1 antibody therapy.

At 4–6 months after the second vaccination, 93.1% (461/495) of patients showed S-IgG antibody concentration exceeding the cut-off value, while the N-IgG positive rate was stable at approximately 1%. The S-IgG positivity rate among the healthy controls was 100% (170/170), and the N-IgG positivity rate was 1.1% (2/170). The S-IgG antibody concentration was still lower among patients with cancer than among healthy controls. The median S-IgG antibody concentration was 198.3 BAU/mL (95% CI of 176.6 to 226.9) among patients and 288.1 BAU/mL (95% CI of 257.5 to 319.7) among the healthy controls.

### Comparison of immunogenicity of COVID-19 vaccines according to different patient factors

The S-IgG concentrations 1–3 months after the second vaccination of patients with cancer were compared among different ages (< 65 and ≥ 65), sexes, BMI levels (< 18.5, normal range, > 30), smoking histories (never smoked, ex-smoker, current smoker), serum albumin levels (less than 3.0 g/dL, 3.0 g/dL or higher), absolute lymphocyte counts (< 500/μL, ≥ 500/μL), and types of COVID-19 vaccines.

**Fig. 4:**
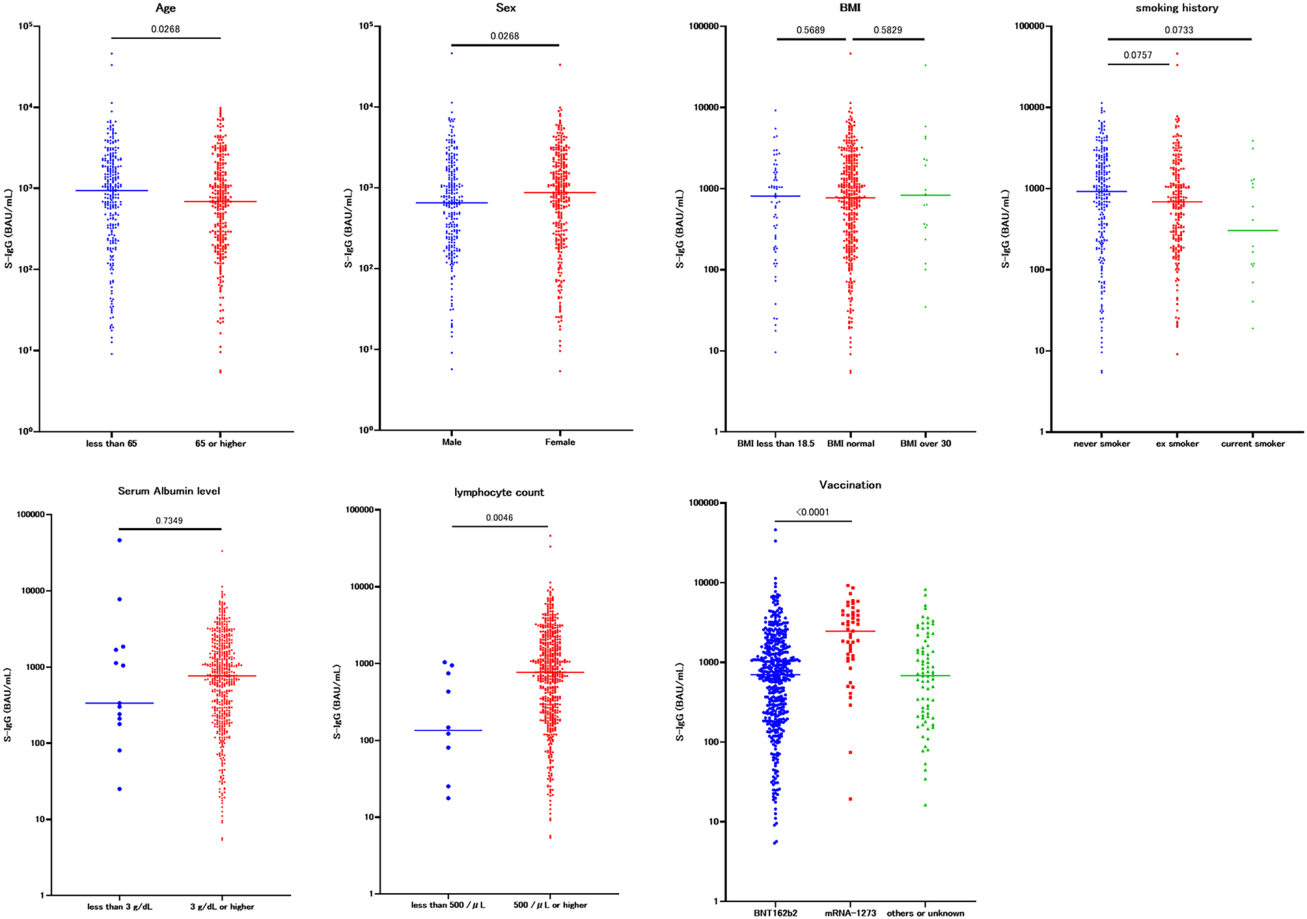
Comparison of immunogenicity of COVID-19 vaccines between different patient factors

The < 65 years group (median 943.2 BAU/mL, 95% CI 757.2–1079) showed significantly high antibody titers compared to the ≥ 65 years group (median 689.8 BAU/mL, 95% CI 579.4–780.9, *p* = 0.0268). The female patients (median 872.9 BAU/mL, 95% CI 736.7–1062) also showed significantly high antibody titers compared to the male patients (median 651.3 BAU/mL, 95% CI 495.6–771.8, *p* = 0.0268). High or low BMI, smoking history, and serum albumin level were not associated with the antibody titer compared to normal BMI. The sufficient lymphocyte count group (500/μL or higher; median 770.8 BAU/mL, 95% CI 687.9–877.2) showed significantly high antibody titers compared to the low lymphocyte count group (< 500/μL; median 134.9 BAU/mL, 95% CI 17.70–947.3, *p* = 0.0046). The mRNA-1273 vaccine group (median 2473 BAU/mL, 95% CI 1645–3408) showed significantly high antibody titers compared to the BNT162b2 vaccine group (median 700.4 BAU/mL, 95% CI 629.1–790.7, *p* < 0.0001) (Fig. 4).

### Impact of cancer treatment on immunogenicity of COVID-19 vaccines

**Fig. 5:**
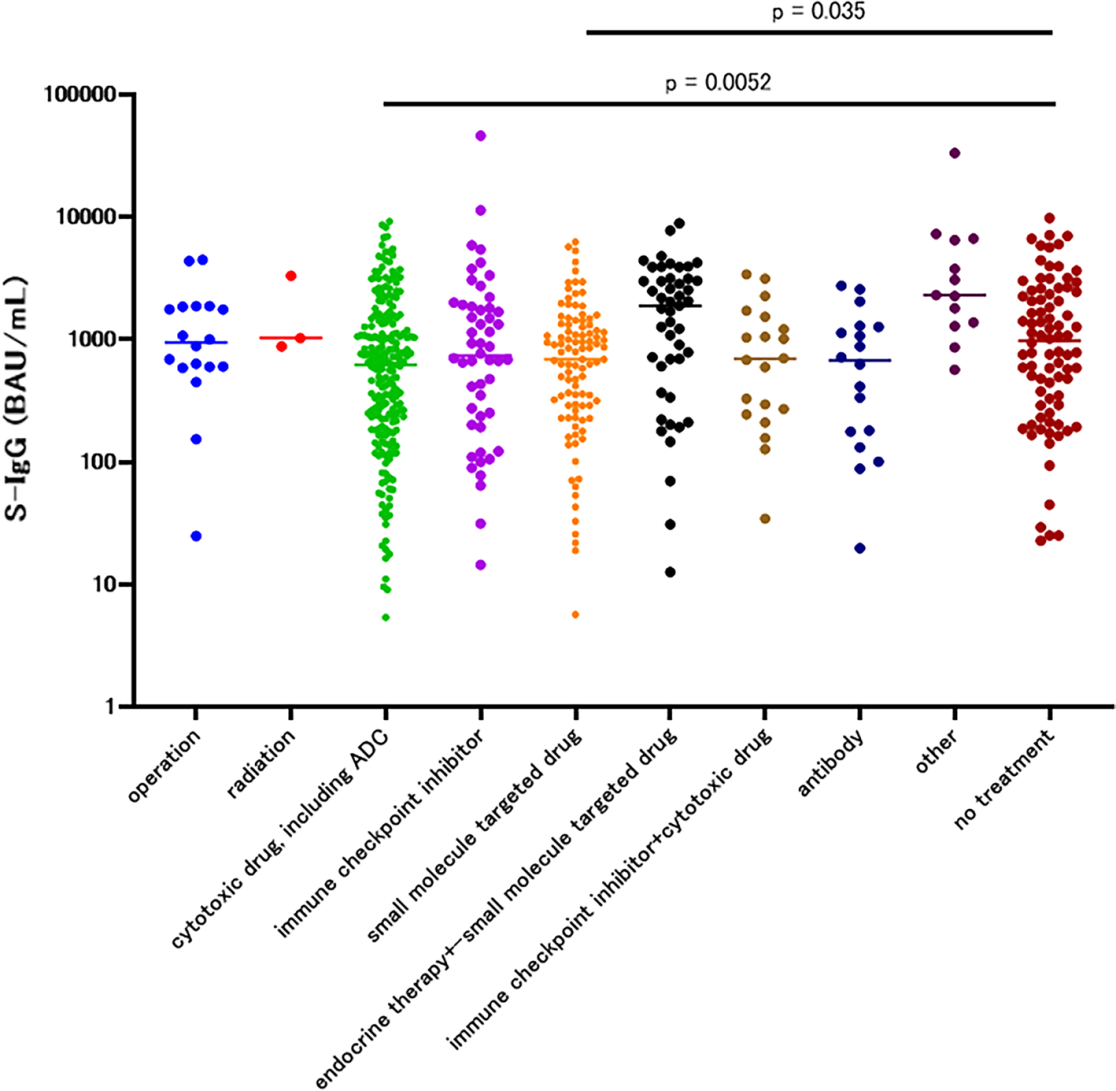
S-IgG of 1–3 months after the second vaccination in each treatment group among the patients

First, the treatment type was classified as cytotoxic drugs (including antibody–drug conjugate); immune checkpoint inhibitor; small molecule drug; hormonal therapy (with or without small molecule targeted drug combination); immune checkpoint inhibitor and cytotoxic drug combination; antibody only; or others. The S-IgG concentration of each treatment type 1–3 months after the second vaccination was compared to the no-treatment group. The cytotoxic drug group (median 623.4 BAU/mL, 95% CI 427.5–757.2, *p* = 0.0052) showed significantly low antibody titers compared to the no-treatment group. The small molecule targeted drug group (median 691.4 BAU/mL, 95% CI 501.4–947.3, *p* = 0.0351) also showed relatively lower antibody titers compared to the no-treatment group, although the difference was not statistically significant (Fig. 5, Table 3a).

**Table 3:** S-IgG concentrations 1–3 months after the second vaccination

(a) In each treatment group among patients										
Treatment	Operation	Radiation	Cytotoxic drug, and antibody drug conjugate	Immune checkpoint inhibitor	Smolecule targeted drug	Endocrine therapy with or without small molecule targeted drug	Immune checkpoint inhibitor + cytotoxic drug	Antibody	Other	No treatment
*N*	18	3	231	52	103	47	20	18	13	85
median (95%CI)	946.5 (601.4–1854)	1031 (881.0–3321)	621.9 (438.6–748.0)	738.4 (432.4–1335)	691.4 (501.4–947.3)	1892 (908.4–2601)	694.6 (270.6–1215)	672.9 (177.2–1270)	2313 (1286–6686)	1021 (592.7–1415)
Mann–whitney	*p* = 0.9338	*p* = 0.5096	*p* = 0.0052*	*p* = 0.3745	*p* = 0.0351	*p* = 0.0847	*p* = 0.3355	*p* = 0.1017		Reference

(b) In each cytotoxic drug group among patients								
Cytotoxic drug	Alkylating agent	Antimetabolite agent	Antimetabolite agent + antimicrotubule agent	Antimetabolite agent + TOPO inhibitor	Antimicrotubule agent	Pt combination	TOPO inhibitor	No treatment
*N*	22	47	14	15	32	88	7	85
Median (95%CI)	57.5 (5.4–748)	765.6 (608.3–1509)	578.8 (81.9–934.9)	757.2 (358.2–1582)	859.5 (345.5–1342)	423.7 (264.2–767.0)	239.1 (69.6–2620)	1021 (592.7–1415)
Mann–whitney	*p* < 0.0001*	*p* = 0.9012	*p* = 0.0129	*p* = 0.6523	*p* = 0.5757	*p* = 0.003*	*p* = 0.2381	Reference

(c) In each molecular-targeted drug group among patients										
Molecular targeted drug	EGFR TKI	CDK4/6	PARP inhibitor	ALK inhibitor	Multi TKI	MAPK inhibitor	mTOR inhibitor	BCR-ABL TKI	Other	No treatment
*N*	37	29	15	13	13	5	5	4	8	85
Median (95%CI)	582 (355.9–906.7)	1393 (908.4–2845)	264 (43.0–690.4)	922.5 (632.1–1608)	1144 (628.0–1939)	1047 (654.2–3639)	227.4 (5.7–323.4)	124.4 (0.0–288.9)	1677 (0.0–5307)	1021 (592.7–1415)
Mann–Whitney	*p* = 0.0649	*p* = 0.1651	p = 0.0004*	*p* = 0.892	*p* = 0.575	*p* = 0.3891	*p* = 0.0036*	*p* = 0.005*		Reference

**Fig. 6:**
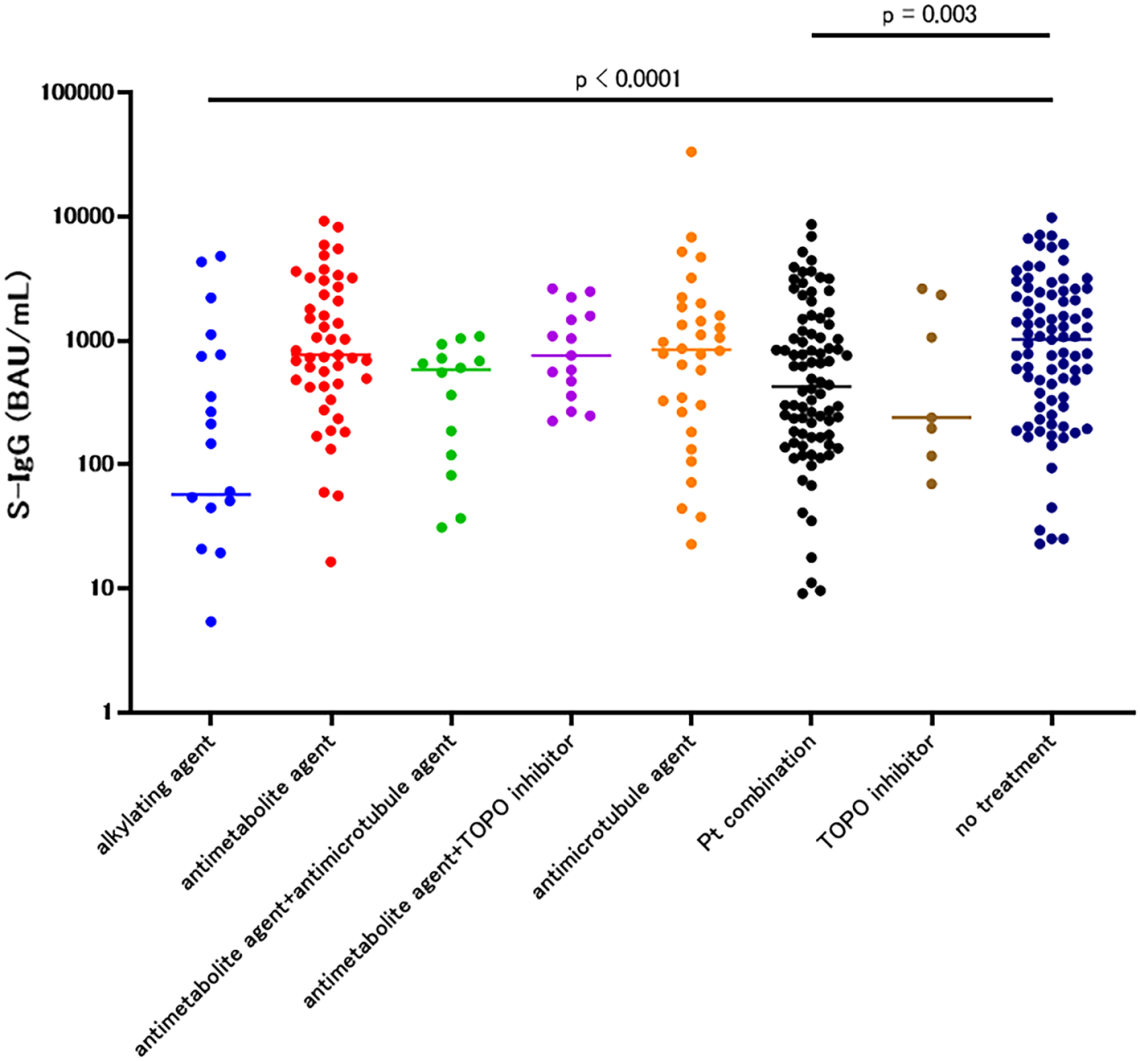
S-IgG of 1–3 months after the second vaccination in each cytotoxic drug group among the patients

**Fig. 7:**
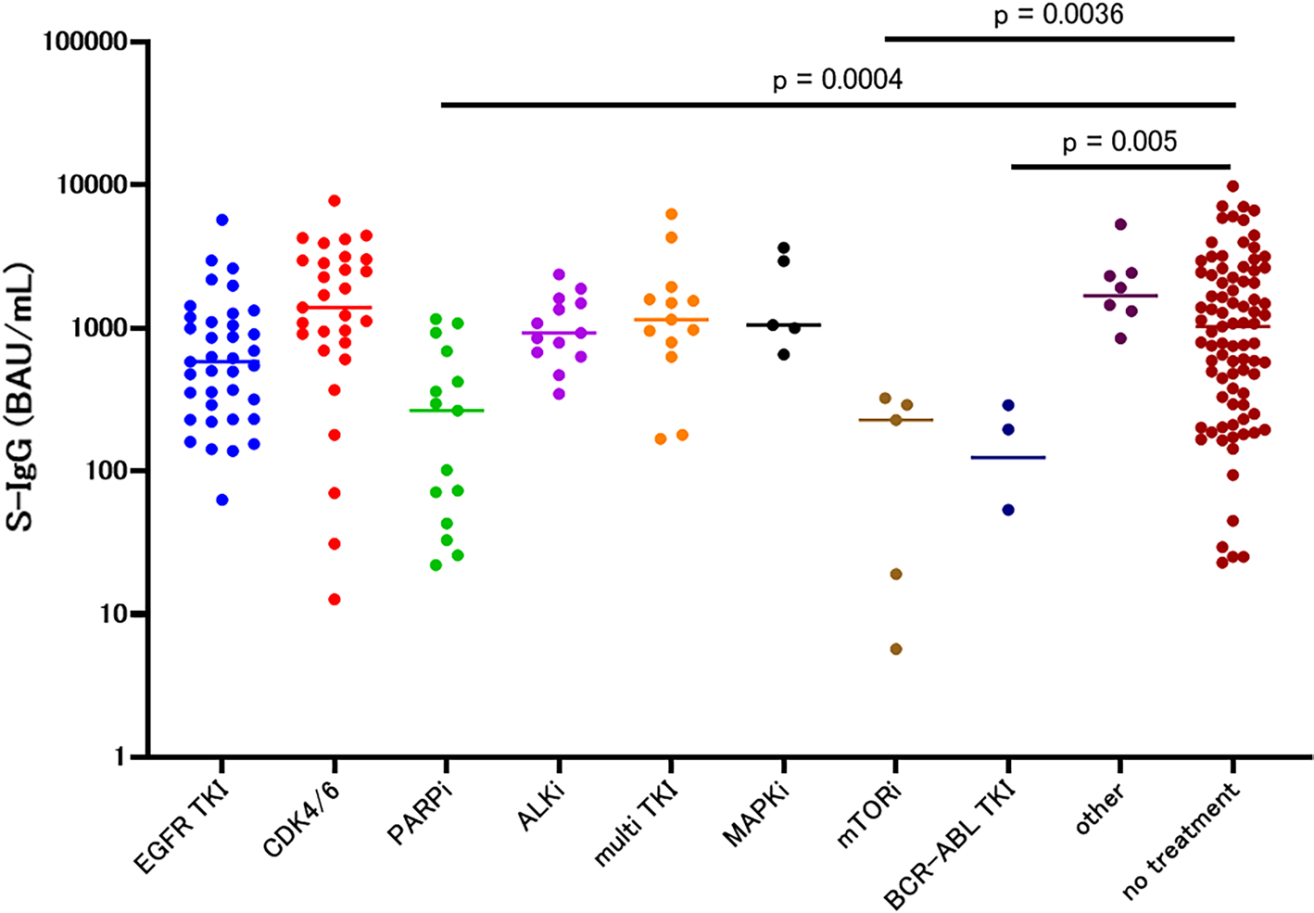
S-IgG of 1–3 months after the second vaccination in each molecular-targeted drug group among the patients

Subsequently, we subdivided treatment types to identify drugs that affected the immune response. Among the cytotoxic drugs, groups treated with alkylating agents (median 57.5, 95% CI 5.4–748, *p* < 0.0001) and platinum agent-containing therapy (median 423.7, 95% CI 264.2–767.0, *p* = 0.003) demonstrated significantly low S-IgG compared to the no-treatment group, while antimetabolite agents, antimicrotubule agents, and topoisomerase inhibitors did not lower the S-IgG value (Fig. 6, Table 3b). Among drugs that target molecules such as EGFR tyrosine kinase inhibitor (TKI), cyclin-dependent kinase 4/6 inhibitor, poly ADP ribose polymerase inhibitor (PARP), anaplastic lymphoma kinase inhibitor, multi TKI, Mitogen-activated protein kinase pathway inhibitor, mammalian target of rapamycin (mTOR) inhibitor, BCR-ABL TKI, and others, the S-IgG concentrations of the PARP inhibitor group (median 264, 95% CI 43–690.4, *p* = 0.0004), mTOR inhibitor group (median 227.4, 95% CI 5.7–323.4, *p* = 0.0036), and BCR-ABL inhibitor group (median 124.4, 95% CI 0.0–288.9, *p* = 0.005) were significantly reduced compared to that of the no-treatment group (Fig. 7, Table 3c).

## Discussion

In our study conducted in a single institution with a large sample size, the seroconversion rate among patients with cancer was lower than that in healthy individuals at both 1–3 months and 4–6 months after the second COVID-19 vaccination. S-IgG antibody concentration among patients was the same level at 1–3 months after the second vaccination, but significantly decreased at 4–6 months compared to healthy individuals. Among patients with cancer, age, sex, lymphocyte count, and vaccination type affected low SARS-CoV-2 S-IgG levels 1–3 months after the second vaccination. SARS-CoV-2 S-IgG levels 1–3 months after the second vaccination were significantly lower in patients who had started certain treatments, such as PARP inhibitor, mTOR inhibitor, BCR-ABL inhibitor, alkylating agent, and platinum combination therapy, at least 1 month prior to vaccination compared to those who had not received any treatment.

There have been few large prospective studies, including the Vaccination Against COVID in Cancer (VOICE) trial in the Netherlands [8], and a comprehensive, longitudinal clinical outcomes and immune profiling program (the CAPTURE study) in the United Kingdom [12], focusing on the immunogenicity of COVID-19 vaccines among patients with cancer. The VOICE trial reported the SARS-CoV-2-binding antibody response in each patient under the active cancer treatment cohort (immunotherapy cohort, chemotherapy cohort, and chemoimmunotherapy cohort) was non-inferior compared with individuals without cancer, but a small proportion of suboptimal and non-responders was detected among patients with solid tumors who were being treated for their cancer. The CAPTURE study revealed 85% and 99% seroconversion rates in patients with solid malignancies and the general population, respectively. Our study showed a similar trend but provided more detailed information on the treatment regimen. An increasing number of small-molecule targeted drugs has been developed for the treatment of cancer, revealing the significance of the different effects of each drug.

Treatment-induced immunosuppression is a plausible explanation of the low immunogenicity of COVID-19 vaccines in patients with cancer. Chemotherapy, such as alkylating agent and platinum-based combination therapies, induced reduction in serologic response due to immunosuppressive effects in addition to the negative effect of glucocorticoids as anti-emetics [13, 14]. mTOR regulates functions of antigen-presenting cells, such as dendritic cells, and plays important roles in the activation of conventional T cells and the function and proliferation of regulatory T cells. Considering the mTOR inhibitor is used as an immunosuppressant drug as well as an anti-cancer drug, the low serologic response is expected. PARP and BCR-ABL inhibitors are known for their potential adverse effect on myelosuppression [15], though laboratory data collected at most one month before vaccination in our study did not show severe reductions in neutrophils or lymphocytes sufficient to explain the low immunogenicity among these patients (data not shown). PARP inhibitors have beneficial effects against SARS-CoV-2 infection through a variety of anti-inflammatory mechanisms including maintaining B cell homeostasis, differentiation, antibody production, and antigen-presenting function of dendritic cell maturation. Although a low SARS-CoV-2 neutralizing antibody level was observed among patients treated with PARP inhibitors, detailed mechanisms for low titers of IgG are unknown [16]. Notably, three out of four patients treated with BCR-ABL inhibitors in our study had solid tumors (gastrointestinal stromal tumors) and low immunogenicity among patients treated with BCR-ABL inhibitors is not attributed to hematologic malignancy. The potential of imatinib for the treatment of COVID-19 has been reported [17]; however, mechanisms for lowering immunogenicity have not been identified.

A limitation of our study is that the serological detection of S-IgG concentration may not necessarily be correlated with functional virus-neutralizing activity, particularly against variants of concern. The dominant variant in Japan was the Delta variant in July 2021, which was replaced by the Omicron variant completely in January 2022 [18]. Secondly, because Japan had a lower incidence of COVID-19 than other countries, it was difficult to compare the COVID-19 infection rate as an endpoint. We were able to estimate the incidence of COVID-19 infection by increased N-IgG concentration—only one patient out of 590 at 103 months after the second vaccination and no patient out of the 495 at 4–6 months after the second vaccination. Third, as the characteristics of patients and hospital staff were quite different, the results of direct comparisons of the two groups could exhibit high ambiguity. Forth, we analyzed the history of treatment of patients before vaccination, but the treatment history after vaccination was not planned to collect. Additionally, we did not collect much data from patients with hematological malignancies in this study because the effect of B-cell-targeted therapies such as anti-CD20 monoclonal antibody has been evaluated in another study on patients with hematological cancer (UMIN000049407).

In conclusion, COVID-19 vaccine immunogenicity was reduced among patients with cancer compared to the healthy controls both 1–3 and 4–6 months after the second vaccination, and several treatment regimens negatively impacted the patients’ immune response.

## Collaborators

Sysmex as co-researcher.
